# Can metabolic prediction be an alternative to genomic prediction in barley?

**DOI:** 10.1371/journal.pone.0234052

**Published:** 2020-06-05

**Authors:** Mathias Ruben Gemmer, Chris Richter, Yong Jiang, Thomas Schmutzer, Manish L. Raorane, Björn Junker, Klaus Pillen, Andreas Maurer

**Affiliations:** 1 Institute of Agricultural and Nutritional Sciences, Chair of Plant Breeding, Martin Luther University Halle-Wittenberg, Halle, Germany; 2 Institute of Pharmacy, Martin Luther University Halle-Wittenberg, Halle, Germany; 3 Department of Breeding Research, Quantitative Genetics, Leibniz Institute of Plant Genetics and Crop Plant Research, Gatersleben, Germany; Institute of Genetics and Developmental Biology Chinese Academy of Sciences, CHINA

## Abstract

Like other crop species, barley, the fourth most important crop worldwide, suffers from the genetic bottleneck effect, where further improvements in performance through classical breeding methods become difficult. Therefore, indirect selection methods are of great interest. Here, genomic prediction (GP) based on 33,005 SNP markers and, alternatively, metabolic prediction (MP) based on 128 metabolites with sampling at two different time points in one year, were applied to predict multi-year agronomic traits in the nested association mapping (NAM) population HEB-25. We found prediction abilities of up to 0.93 for plant height with SNP markers and of up to 0.61 for flowering time with metabolites. Interestingly, prediction abilities in GP increased after reducing the number of incorporated SNP markers. The estimated effects of GP and MP were highly concordant, indicating MP as an interesting alternative to GP, being able to reflect a stable genotype-specific metabolite profile. In MP, sampling at an early developmental stage outperformed sampling at a later stage. The results confirm the value of GP for future breeding. With MP, an interesting alternative was also applied successfully. However, based on our results, usage of MP alone cannot be recommended in barley. Nevertheless, MP can assist in unravelling physiological pathways for the expression of agronomically important traits.

## Introduction

Barley (*Hordeum vulgare* L.) is the fourth most important crop worldwide after wheat, maize and rice, with an acreage of 48.1 m hectares in 2017/18 [1]. Approximately 10,000 years ago, barley was domesticated and is thus one of the oldest crop plants [2]. Domestication and breeding for yield performance in elite barley (*Hordeum vulgare* ssp. *vulgare*) led to a reduction of biodiversity through allele erosion, the so-called genetic bottleneck effect. This phenomenon also applies to most other crop species [3, 4]. Consequently, further improvement of the performance of barley becomes increasingly difficult. Moreover, classical selection methods with several years of field trials are expensive. On top of that, the current climate change scenarios and the increasing world population, pose difficult challenges for breeders to use effective breeding methods that could lead to yield increase and stability.

To accelerate the breeding progress, indirect selection methods are of great importance. The most common method is the single nucleotide polymorphism (SNP) based estimation of breeding values through genomic prediction (GP) [5]. The advantage of GP is the early estimation of agronomically relevant traits already at seedling stage of single plants, which accelerates the selection of the best plants during the breeding process. In contrast to classical methods like genome-wide association studies (GWAS) or linkage mapping to define trait-specific molecular markers for subsequent marker-assisted selection (MAS), the approach of GP (also called genomic selection–GS) is different: Rather than focusing on the single effect and the position of one marker, the entirety of all markers is taken into account in GP. Ordinarily, for each marker allele, an effect is estimated and with the combination of all marker effects, a genomic estimated breeding value (GEBV) is computed. Depending on the model, interactions between alleles may also be included in the calculation of GEBV. This requires a large number (tens or hundreds of thousands) of markers distributed over the whole genome [6, 7]. The modern methods of genome sequencing and the large number of genotyped SNPs allow a broad application of GP across different living systems—including animal and plant species as well as human genetics [8]. GP overcomes the disadvantages of MAS, which mainly relies on few selected quantitative trait loci (QTL), identified through linkage mapping and GWAS. Those methods have achieved great success, also in barley, for instance in the elucidation of genetic issues like disease resistance and flowering [9–11]. However, the classical methods have certain weaknesses in the quantification of some polygenic traits that are influenced by numerous minor QTL with small effects [12]. This circumstance is considered in GP by assigning effects to all markers tested.

Apart from GP, studies with different species (*Arabidopsis thaliana*, tomato, rice, potato, maize) confirmed that a reliable estimation of trait performance is also possible through MP with metabolite data [13–17]. Metabolites play an important role in all living organism, so in plants. Estimates for the total number of metabolites in plant kingdom vary from 200,000 to 1,000,000 [18]. A rough classification of metabolites is the differentiation between primary and secondary metabolites. While the primary metabolites are responsible for growth and development, the secondary ones are built in response to various biotic and abiotic stresses. These two classes are subject of different genetic control. Whereas primary metabolites are mainly controlled by many interacting genes with small effects, the secondary ones are determined by a small number of genes with large effects [19–22]. The use of metabolite profiling in plant breeding is interesting as it can provide helpful information about the system under study; metabolites play a key role in gene expression and help to elucidate the function of genes [23]. Furthermore, metabolites can be used as biomarkers (when no genomic information is available) or as an addition to SNP markers to predict phenotype expression [16]. With a combination of gas chromatography and mass spectrometry (GC-MS), a high-throughput method for untargeted metabolite screening is available. Other high-throughput methods such as liquid chromatography-mass spectrometry (LC-MS) and nuclear magnetic resonance-mass spectrometry (NMR-MS) have also been established for metabolite profiling of the experimental system [24].

In this project we simultaneously characterize the multi-parental wild barley nested association mapping (NAM) population HEB-25 [11] with SNPs (50k SNP array [25]) and through metabolic profiling of 128 metabolites with sampling at two different developmental stages. We merge SNP, metabolite and phenotype data to alternatively predict phenotypes based on metabolites, SNPs or a combination of both and compare the prediction accuracies of the different methods.

## Materials and methods

### Plant material

The population HEB-25 is the worldwide first NAM population of barley. It was generated by crossing and subsequent backcrossing of 25 wild barley accessions (24 *Hordeum vulgare* ssp. *spontaneum* and one *Hordeum vulgare* ssp. *agriocrithon*) with the German elite spring barley cultivar Barke (*Hordeum vulgare* ssp. *vulgare*). The resulting BC_1_S_3_ generation comprises 1,420 individual lines (whereof 1,307 were used in this study) subdivided into 25 families (for a detailed description see [11]).

### Genotypic evaluation

DNA of pooled BC_1_S_3:8_ plants of each line was extracted according to the manufacturer’s protocol, using the BioSprint 96 DNA Plant Kit and a BioSprint work station (Qiagen, Hilden, Germany), and finally dissolved in distilled water at approximately 50 ng/μl for genotyping with the recently developed barley Infinium iSelect 50K chip [25] at TraitGenetics, Gatersleben, Germany. SNP markers that did not meet the quality criteria (polymorphic in at least one HEB family, < 10% failure rate, < 12.5% heterozygous calls) were removed from the data set. Altogether, 33,005 SNPs met the quality criteria and were analysed in this study. Based on the Barke reference genotype, the wild barley allele can be specified in each segregating family. To set up the quantitative identity-by-state (IBS) matrix the state of the homozygous Barke allele was coded as 0, while HEB lines that showed a homozygous wild barley genotype were assigned a value of 2. Consequently, heterozygous HEB lines were assigned a value of 1. If a SNP was monomorphic in one HEB family but polymorphic in a second family, lines of the first HEB family were assigned a genotype value of 0, since their state is not different from the Barke allele. Gaps resulting from missing genotypes (0.84%) were estimated by applying the mean imputation (MNI) approach [26]. The genotype matrix is available at e!DAL [27, 28]. The markers are uniformly distributed over the whole genome with few gaps and decreasing density in the telomere regions (S1 Fig).

### Field trials

Between 2011 and 2018, eight field trials with HEB-25 were conducted at the Kühnfeld experimental station of the University Halle (51°29'45.72"N; 11°59'36.62"E) to gather phenotypic data. All field trials were sown in spring between March and April with fertilisation and pest management following local practice. Detailed information about field trials is given in S1 Table and S1 File.

The studies were conducted on land owned by the authors’ institutions. The research conducted complied with all institutional and national guidelines.

### Phenotypic evaluation

The following traits were measured in the field trials: time to shooting (SHO), flowering (HEA) and maturity (MAT); plant height (HEI); number of ears per m^2^ (EAR); grain number per ear (GNE); thousand grain weight (TGW); grain yield (YLD). Table 1 shows a detailed description of the trait assessment. Raw phenotype data is available as S2 File.

**Table 1:** List of evaluated traits for HEB-25 in eight-year field trials.

Abbr.^a^	Trait	Unit	Method of measurement	Years studied
SHO	Time to shooting	days	Number of days from sowing until first node noticeable 1 cm above soil surface for 50% of all plants of a plot, BBCH 31 [29].	2011–2018
HEA	Time to heading (flowering)	days	Number of days from sowing until emergence of 50% of ears on main tillers of a plot, BBCH 49 [29].	2011–2018
MAT	Time to maturity	days	Number of days from sowing until hard dough: grain content firm and fingernail impression held, BBCH 87 [29].	2012–2018
HEI	Plant height	cm	Average plant height of all plants of a plot measured from soil surface to tip of the erected ear without awns at maturity.	2011–2018
EAR	Ears per m^2^	n	Number of ears per m^2^, counted in a representative 50 cm frame in the middle of a row and extrapolated to one m^2^.	2014–2018
GNE	Grain number per ear	n	Number of grains per ear, calculated by use of MARVIN seed analyser (GTA Sensorik GmbH, Neubrandenburg, Germany) based on a representative sample of 10 ears.	2014–2018
TGW	Thousand grain weight	g	Weight of 1000 grains, calculated after harvest by use of MARVIN seed analyser based on a 200 seeds sample of each plot (2011–2013). Before, seeds were cleaned and damaged seeds were sorted out.	2011–2018
YLD	Grain yield	dt/ha	Total grain yield determined after harvest of the whole plot (2014–2016) or derived from the three yield components EAR, GNE and TGW (2017/18) and extrapolated to dt/ha.	2014–2018

^a^ Abbreviations of traits.

### Metabolic evaluation

A 2 cm tissue sample from the middle region of the last fully developed leaf of each HEB line was sampled on 22 May 2017 under a clear sky between nine and ten o’clock. This date represented the developmental stage BBCH 30–31 (beginning of shooting) for the majority of plants. The leaf was cut approximately 1 cm from the stem and was put in an Eppendorf tube. The protruding leaf was cut off, the Eppendorf tube was closed and put instantly in liquid nitrogen to stop metabolic processes. All plots were sampled within one hour under constant weather conditions. In total, 29 people were involved to meet this schedule. Sampling was repeated under the same circumstances (constantly clear sky, equal time of day, equal sampling methods) on 22 June 2017. The plants were more heterogeneous at this time, representing developmental stages BBCH 59–69 (end of ear emergence to end of flowering).

The frozen leaf samples were pulverised using a Retsch-ball mill (MM 400, Retsch, Germany) for 2 minutes at 20 Hz. The homogenised leaf samples were then resuspended in 700 μl methanol:chloroform:water solution (3:2:4) containing 8 μg/ml ^13^C-sorbitol as an internal quantitative standard. The mixture was shaken for 20 min at room temperature and at 500 rpm. The mixture was then centrifuged for 11,000 X g for 5 minutes at 4°C. After the extraction, 10 μl of the supernatant was dried in a vacuum concentrator without heating for 45 minutes. Online derivatization was performed using the Multi-Purpose Sampler (MPS, Gerstel, Germany) by adding 30 μl Methoxamine hydrochloride (20 mg/ml in Pyridine) to the samples and shaken for 30 min at 45°C. Furthermore, 45 μl N,O-Bis(Trimethylsilyl)trifluoroacetamide and 5 μl Alkane-Standard (C10-C28; 6 mg/ml) were added and the samples were shaken again for 120 min at 45°C. As quality controls for the extraction procedure, leaf samples from 10 randomly chosen Barke reference lines were extracted and pooled together. All the samples along with 20% of quality controls were analysed with GC-MS (GC-qTOF system -7890B/7200, Agilent, Santa Clara, USA). One μl of the derivatized samples were injected at 250°C in a splitless mode with a helium gas flow set to 1 ml min^-1^. Chromatography was performed with a 30-m Zebron Capillary GC-Column (ZB-Semi Volatiles, 30 m, 0.25 mm, 0.25 μm). The Helium flow was constant at 1 ml/min. The temperature program was set to 60°C followed by a linear ramp of 10°C/min to 320°C and holding at this temperature for 3 minutes. Throughout the run, the transfer line, source and the quadrupole were set to 290°C, 230°C and 150°C respectively.

The raw data was processed by MassHunter Qualitative Analysis software (Agilent, B.07.00) and MassHunter Quantitative Analysis software for QTOF (Agilent, B.08.00). The mass spectra library NIST 14 (National Institute of Standards and Technology) and standard compounds were used for identification and confirmation of the chromatographic peaks. Peak areas were normalized with the internal standard and fresh weight.

This resulted in data for 1,307 lines where 158 metabolites (alkanes, amino acids, organic acids, sugars and unknowns) could be defined (S3 File). Metabolites with > 10% missing values were removed from the data set so that 128 metabolites were used for prediction (S2 Table). Samples from the 2^nd^ sample date resulted in data for 1,229 lines with 159 metabolites (one additional unknown metabolite). After data cleaning 122 metabolites remained for the subsequent analyses (S3 Table). Remaining missing values were replaced with the minimum value of the respective metabolite.

### Statistical analyses

All statistical analyses were performed with SAS 9.4 [30] and R [31]. Broad-sense heritabilities were computed using R software with the lmerTest package [32] across treatments and years as $h^{2}=\frac{V_{G}}{V_{G}+\frac{V_{GY}}{y}+\frac{V_{R}}{yr}}$, where V_G_, V_GY_ and V_R_ represent the genotype, genotype ⨯ year, and error variance components, respectively. The terms y and r indicate the number of years and replicates, respectively. To estimate variance components, all effects were assumed to be random. Best linear unbiased estimates (BLUEs) of all traits were calculated using the PROC HPMIXED procedure in SAS for each genotype assuming fixed genotype effects. Pearson’s correlation coefficients were calculated with R software with the corrgram package [33]. The box-cox power transformation [34] was applied to metabolic data using SAS PROC TRANSREG with λ ranging from -3 to 3 by steps of 0.25. The genomic heritabilities of metabolites (also called SNP-based heritabilities, [35]) were estimated with the R package sommer [36] as $h_{SNP}^{2}=\frac{\sigma_{A}^{2}+\sigma_{D}^{2}+\sigma_{I}^{2}}{\sigma_{A}^{2}+\sigma_{D}^{2}+\sigma_{I}^{2}+\sigma_{R}^{2}}$, where $\sigma_{A}^{2},\;\sigma_{D}^{2},\;\sigma_{I}^{2}$ and $\sigma_{R}^{2}$ represent the additive, dominance, epistatic and residual variance components, respectively. Additionally, repeatability of metabolites was calculated as $rep=\frac{V_{G}}{V_{G}+\frac{V_{R}}{r}}$ for the subset of 17 genotypes (elite cultivars, control lines) where multiple metabolite measurements were available. Euclidean distance matrices with SNP and metabolite data were calculated using R package stats. Subsets of SNPs or metabolites for GP and MP were created using R package dpylr [37]. Descriptive statistics for metabolites were calculated with R package psych [38]. Two-sided t-tests were carried out to detect significant differences between the models and datasets. The significance level was set to p < 0.01. All figures were created with R using the package ggplot2 [39].

### Genomic/metabolic prediction

Based on BLUEs of the 1,307 HEB genotypes (1,307 lines with complete datasets of SNP and metabolite data at the 1^st^ sampling date, 1,229 lines at the 2^nd^ sampling date), two approaches for genomic prediction were applied considering additive effects: ridge regression best linear unbiased prediction (RR-BLUP) [40] and BayesB [41]. All statistical procedures for genomic prediction approaches were executed using R. The R code for RR-BLUP was developed in-house [42]. For the BayesB model, the package BGLR [43] was used. The models are briefly described in the following.

Let n be the number of genotypes, m be the number of markers and l be the number of years. The RR-BLUP model has the form ***y*** = **1***_n_μ*+***Xg+e***, where ***y*** is the vector of BLUEs of the respective trait for all HEB genotypes across years, **1**_*n*_ denotes the vector of 1’s, *μ* is the common intercept term, ***g*** = (*g*_1_,*g*_2_,…,*g_m_*)′ is the vector of marker effects, ***X*** is the matrix of marker information and ***e*** is the residual term. In the model we assumed that $g∼N(0,\sigma_{g}^{2}I)$, $e∼N(0,\sigma_{e}^{2}I)$, where σ^2^_g_ = σ^2^_G_ / m for SNP markers and σ^2^_e_ = σ^2^_R_ / l. Here σ^2^_G_ and σ^2^_R_ are the genotypic and residual variance components obtained in the mixed model in the phenotypic data analysis. The penalty parameter is λ = (σ^2^_R_ / l) / (σ^2^_G_ / m). The estimation of marker effects is then given by the mixed model equations [44]. The basic model of BayesB is the same as RR-BLUP. However, all parameters are treated as random variables in a Bayesian framework and we do not assume the same variance for all marker effects. More precisely, we defined the prior distributions as $g∼N(0,D),e∼N(0,\sigma_{e}^{2}I)$, where $D=diag(\sigma_{g_{1}}^{2},\sigma_{g_{2}}^{2},\ldots ,\sigma_{g_{p}}^{2})$. For the intercept term *μ* we assume a flat prior. For each i, the prior distribution of $\sigma_{g_{i}}^{2}$ is assumed to be zero with probability π and a scaled inverse chi-squared distribution with probability (1-π). The prior of π is a beta distribution. The prior of σ^2^_e_ is also a scaled inverse chi-squared distribution. A Gibbs sampler algorithm was then applied to infer all the parameters in the model.

The accuracy of the prediction by the models was evaluated using five-fold cross-validation [45]. In each run of cross-validation, the training set included 80% of HEB lines, randomly selected per HEB family, while the remaining 20% of HEB lines were assigned to build the test set. The prediction ability (r_ab_) is the correlation between observed and predicted values, averaged over all 100 cross-validation runs. Prediction accuracy (r_ac_) is defined as $r_{ac}=\frac{r_{ab}}{\sqrt{h^{2}}}$ [17]. Pairwise t-tests were carried out in R to determine significant differences in prediction accuracy between models and prediction methods. The significance level was set to p < 0.01.

Genomic prediction was realised for the agronomic traits measured in the field with 33,005 SNPs coded as 0,1,2 in the RR-BLUP model and -1,0,1 in the BayesB model to meet the specific requirements of the applied R packages.

For metabolic prediction (MP) of the agronomic traits measured in the field, the values of 128 metabolites (first sampling date) or 122 metabolites (second sampling date) were used in both models. In the combined approach, all 33,005 SNPs and 128 metabolites (or 122) were included in the prediction model.

## Results and discussion

### Phenotypic data

Descriptive analysis of the phenotypic data showed a high variation between lines and between years, resulting in high coefficients of variation (S4 Table). For instance, the difference for the trait HEA was 71 days between the minimum and maximum value. This reflects the high diversity of the HEB-25 population within and across years (S2 Fig). Heritabilities for all traits calculated over 4–8 years were > 0.8 with the exception of EAR (0.41) and YLD (0.58, Table 2). In summary, this reflects the high quality of phenotypic data and the genotype impact on traits, underlining the suitability for genetic analyses such as GP and MP.

**Table 2:** Summary of genomic and metabolic prediction, BayesB model.

		SNPs			Metabolites			SNPs + Metabolites			
Trait	h^2^	r_ab_	r_ac_	SD	r_ab_	r_ac_	SD	r_ab_	r_ac_	SD	Sig.
SHO	0.91	0.88	0.93	0.02	0.57	0.59	0.05	0.89	0.93	0.02	-
HEA	0.93	0.87	0.91	0.02	0.59	0.61	0.05	0.88	0.91	0.02	-
MAT	0.83	0.87	0.95	0.02	0.55	0.61	0.06	0.88	0.96	0.01	*
HEI	0.91	0.93	0.97	0.01	0.37	0.39	0.05	0.93	0.97	0.01	-
EAR	0.41	0.74	1.14	0.04	0.38	0.59	0.09	0.74	1.15	0.04	-
GNE	0.84	0.88	0.96	0.04	0.27	0.29	0.07	0.88	0.96	0.03	-
TGW	0.83	0.86	0.94	0.02	0.26	0.28	0.06	0.86	0.94	0.02	-
YLD	0.58	0.77	1.01	0.03	0.35	0.46	0.08	0.77	1.01	0.03	-

BayesB model was applied using SNP and/or metabolite data. Prediction abilities (r_ab_) and prediction accuracies (r_ac_) for selected traits, averaged over 100 cross-validation runs and the standard deviation of r_ac_ (SD) are shown. In addition, heritabilities (h^2^) for the traits are given. Sign. indicates the significance (*: p < 0.01, -: not significant) of a t-test between r_ac_ of SNPs and r_ac_ of SNPs + metabolites for the respective traits. Not indicated here: SNPs and SNPs + metabolites generally performed significantly better than metabolites alone for all traits. Trait abbreviations are given in Table 1.

### Genomic and metabolic prediction

**Fig 1:**
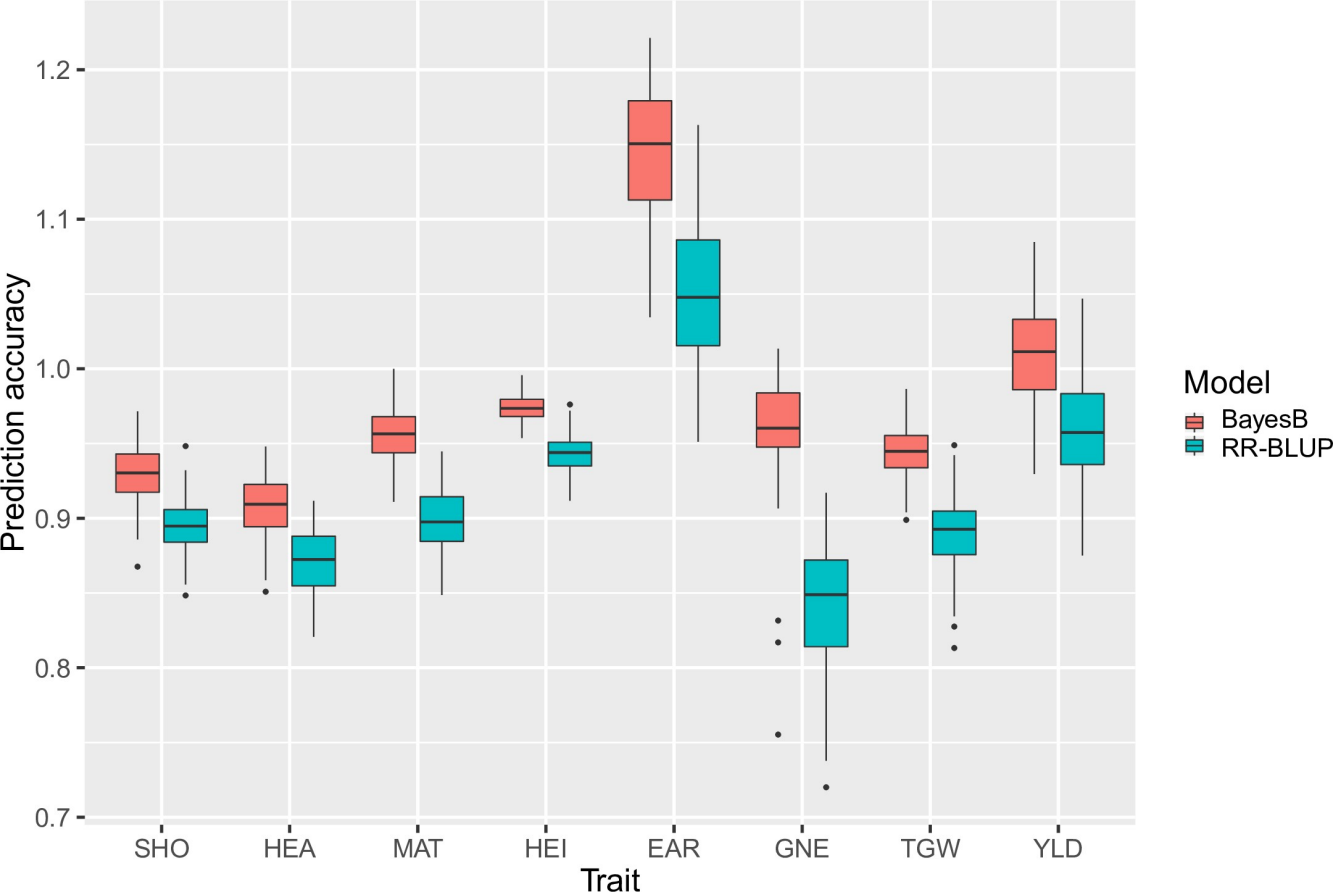
Cross-validated prediction accuracies of traits with SNP data using RR-BLUP and BayesB, respectively. Boxplots contain all 100 prediction values of the cross-validation runs. Red boxes show results of BayesB, while blue boxes show results of RR-BLUP. BayesB performed better for all traits. Prediction accuracies with BayesB were significantly better than with RR-BLUP for all traits.

All results described below (including figures, tables and supplementary files) refer to the metabolite set of the first sampling date unless it is mentioned otherwise. Generally, in genomic prediction with SNP data, we observed a slight advantage of BayesB over RR-BLUP regarding prediction performance, which was significant for all traits (Fig 1). With metabolite data both models performed almost equal (S5 Table, S3 Fig). With the exception of EAR (better performance of RR-BLUP) and YLD (better performance of BayesB), no significant differences were detected. The better performance of BayesB depends on the genetic architecture of the target trait [46]. It is superior to RR-BLUP when the trait is controlled by few large QTL effects, which is true and well-studied for HEA [11] as well as for GNE and TGW [47] in the HEB-25 population. With SNP data high prediction accuracies (≥ 0.91) for all traits were reached with BayesB (Table 2). It is noticeable that the accuracies for the traits EAR and YLD were > 1, which is caused by the low h^2^ estimates of these traits. Nevertheless, the usage of r_ac_ is common in GP, as it corrects r_ab_ for nongenetic effects of the target trait [17]. The correlation between h^2^ and r_ab_ was highly positive (r = 0.95) and, consequently, the correlation between h^2^ and r_ac_ was highly negative (r = -0.94). This underlines the importance of high-quality phenotypic data, resulting in high prediction performance. The observed prediction accuracies are comparable to other studies in wheat, maize and barley [8, 17, 48].

The concept of estimating SNP-based heritability [35], also called genomic heritability, was applied to the metabolite data resulting in values of up to 0.50 with a mean value of 0.10 (S6 and S7 Tables, S4 Fig). Repeatabilities of metabolite measurements showed high variation across metabolites (0.00–0.87) with a mean value of 0.26 (S6 and S7 Tables), hinting on limited data quality for several metabolites that may affect metabolic prediction.

Prediction accuracies with metabolite data instead of SNPs were generally lower. The highest accuracies were observed for the developmental parameters (r_ac_ up to 0.61 for HEA and MAT), while for HEI and especially the yield parameters GNE and TGW low accuracies of no more than 0.29 were obtained (Table 2). The decay of r_ac_ for yield parameters seems logical since sampling took place early on during the shooting phase of plants. The assumption is that metabolites which are involved in plant development are more reflected in the early metabolite profile than the ones responsible for grain filling and yield formation and vice versa. To pursue this question, it is worth to compare r_ac_ of the first sampling with r_ac_ of the second sampling (S8 Table). Actually, based on the second metabolite sampling the prediction accuracies for developmental traits were worse (ca. 0.10 less for SHO and HEA), but also for yield parameters no notable improvements could be achieved. Metabolic prediction with data from the first sampling date performed significantly better for the traits SHO, HEA and HEI. MAT and EAR showed no significant differences. Slight, but significant improvements at the second sampling date could be achieved for the yield parameters GNE, TGW and YLD. In conclusion, sampling during a young and more homogeneous plant stage seems more effective, also in terms of time management.

To our knowledge, there exists no study on MP in barley. Prediction accuracies of MP were, depending on the trait, below the accuracies reported in studies with other species [15–17]. However, the comparability of different studies on MP is difficult, since metabolite determination is highly sensitive. Steinfath et al. [16] predicted blackspot susceptibility of potatoes with correlations between observed and predicted values ranging from 0.68 to 0.82. Riedelsheimer et al. [17] reached accuracies of up to 0.80 for female flowering in maize. The use of both SNPs and metabolites in the combined approach did not lead to an improvement in prediction compared to the sole use of SNPs. This applies to our study as well as to Riedelsheimer et al. [17].

To gain insights which metabolites are decisive for different trait predictions, Pearson’s correlations between metabolite measurements and agronomic traits across all lines were calculated. As expected, correlations were comparably low (-0.36 < r < 0.30, S9 Table), showing that single metabolites generally exert only a moderate impact on trait expression. Interestingly, one of the strongest negative correlations was observed for TMET101 and HEA (*r* = -0.35), indicating that this unknown metabolite might be directly involved in flowering time regulation. This is confirmed by the high effect estimation for TMET101 in the MP model for HEA (S9 Table). In general, there was the trend that metabolites with a high effect estimated in MP also had a higher correlation with the respective agronomic trait, as exemplified for HEA (S5 Fig). Similar observations could be made in the metabolite set of the second sampling (S10 Table). This indicates that MP effect estimates can give hints to metabolites that are involved in trait expression and thus might be worth further investigation for instance to deepen the understanding of molecular pathways.

The accuracies with metabolite data seem to be low compared to the accuracies with SNP data. However, it is important to remember that 128 metabolites face 33,005 SNPs (approximately 260 times more SNPs). Moreover, metabolites were sampled in an early developmental stage of the plants, reflecting just a snapshot in the highly dynamic system of plant metabolism, and used for prediction of eight-year phenotypic data. This raises the question of whether the metabolites are used to predict something they cannot provide. Therefore, the MP model was run again, restricting the phenotypic data to the season 2017, the year in which also the metabolite samples were collected. Surprisingly, this resulted in almost equal or even slightly lower prediction accuracies compared to eight-year phenotypes (S11 Table). With r_ac_ = 0.47 for MAT, the prediction accuracy was even worse. However, the metabolite-trait correlations were quite similar to the complete set (S12 Table). Like SNPs, metabolites seem to fix information about the underlying genotype, which seems to be environmentally stable. Our results support the assumption that a prediction of phenotypic traits is possible even with metabolite data from one year at one sampling date.

A closer look on the estimated effects in GP and MP showed that there was a clear correlation pattern between the estimated effects of different traits (S6A and S6B Fig). Both in GP and MP, the marker and metabolite effects for SHO, HEA and MAT were highly correlated (0.88 < r < 0.95), indicating that the same genes and metabolites are responsible for the expression of these traits. Interestingly, the correlation plot of the phenotypic traits (S6C Fig) reflected the same patterns like the plots for the estimated effects of GP and MP. For instance, the negative correlations between TGW and the developmental parameters (-0.22 < r < -0.37) were quite close to the correlations of their estimated effects, the same applies to the correlations among developmental parameters. Apparently, the GP and MP models were able to quantify these phenotypic connections in their estimation of effects with high precision and therefore they reflected the underlying genetic and metabolic mechanisms. Remarkably, the genetic and metabolic distance matrices were not correlated (r = 0.04, S7 Fig). It seems that they contain similar information, though based on different backgrounds.

**Fig 2:**
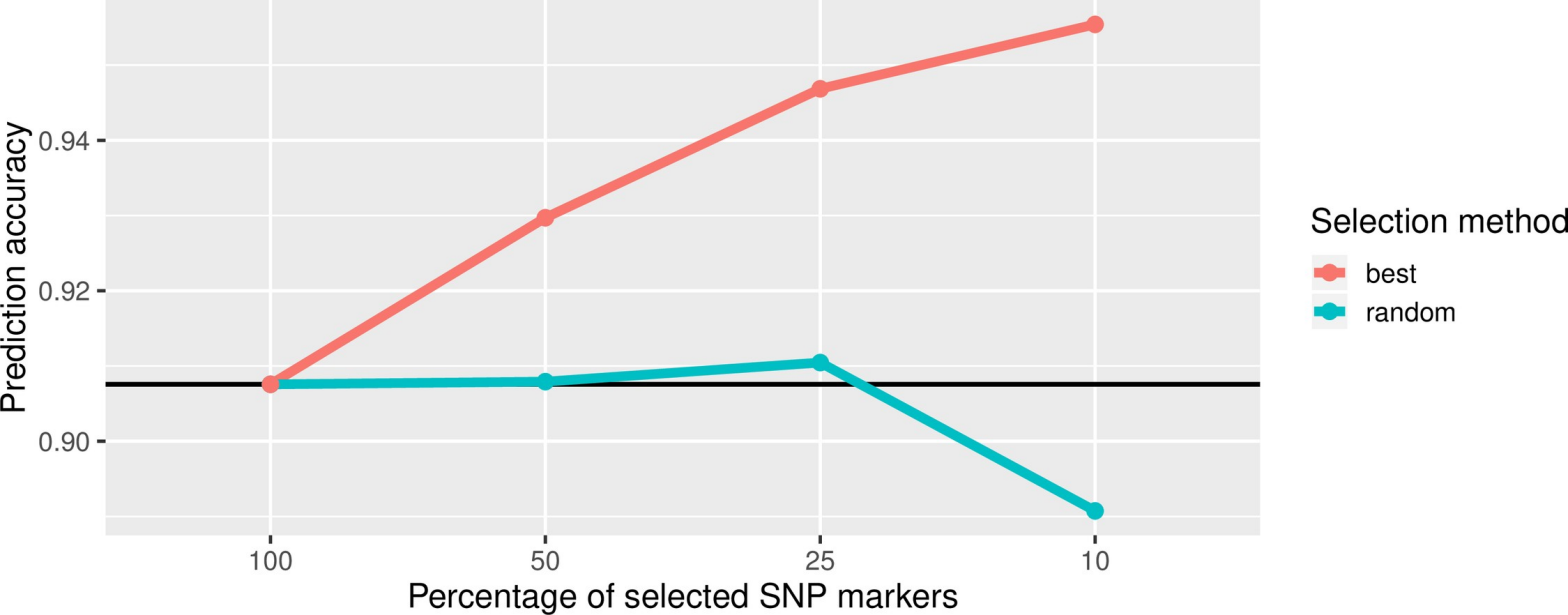
Variation of prediction accuracy for HEA with BayesB after reduction of the number of SNP markers. The black reference line indicates the prediction accuracy using all SNP markers in the model. The red line indicates the trend of prediction accuracy by selecting the best markers (markers with the highest effects in BayesB model), the blue line indicates the trend of prediction accuracy by selecting random markers.

Interestingly, a reduction of used SNPs and metabolites in the prediction model can lead to an improvement or at least to no decay in prediction accuracy. For instance, the prediction accuracy for HEA was steadily increased when reducing the number of SNP markers to subsets of 50%, 25% and 10%, provided that the markers with the biggest effects in GP from the model with the whole marker set were selected. But even with 25% randomly selected markers (8,251 SNP markers) of the complete set a small increase in r_ac_ was observed (Fig 2). Selecting the best markers increased the r_ac_ for all investigated traits whereas random selection, especially by selecting only 10%, clearly reduced the accuracy (S8 Fig). The reason for enhanced prediction accuracy with best markers may be the reduction of SNPs causing background noise in the model. But even random selection did not worsen the accuracy up to a certain point suggesting that fewer markers are sufficient for a reliable coverage of genome information.

**Fig 3:**
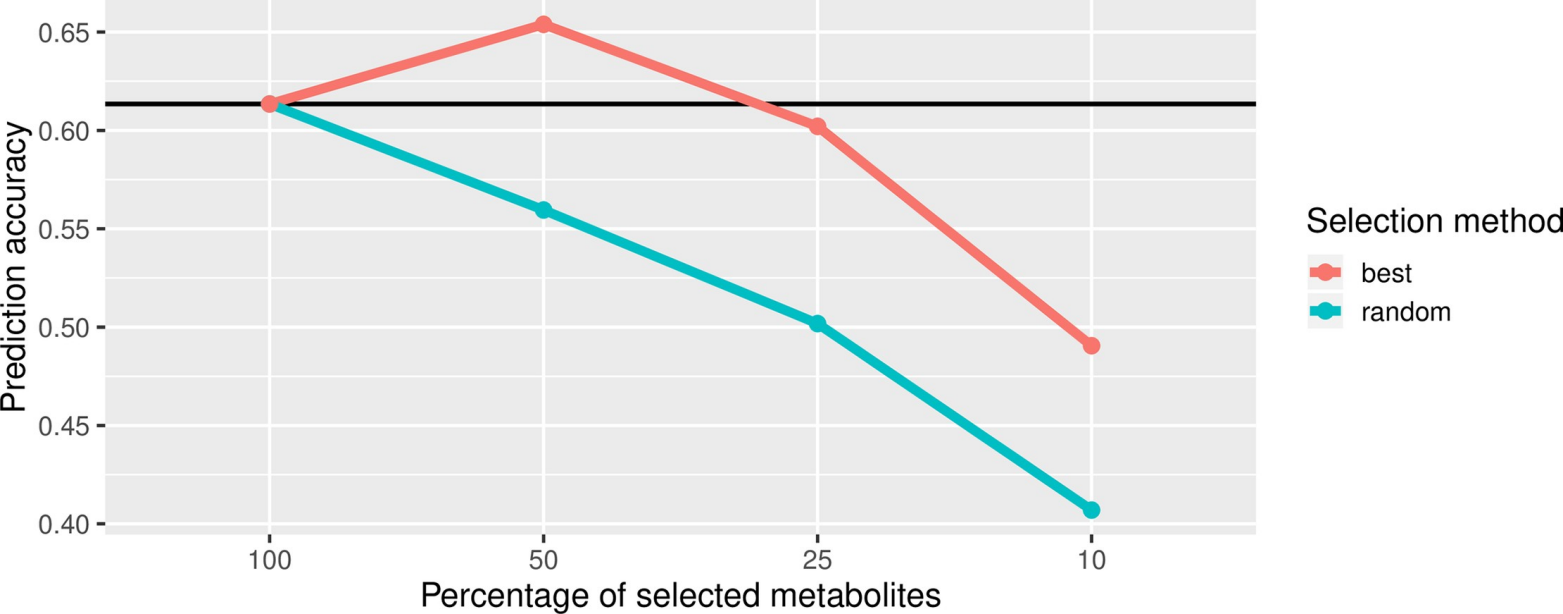
Variation of prediction accuracy for HEA with BayesB after reduction of the number of metabolites. The black reference line indicates the prediction accuracy using all metabolites in the model. The red line indicates the trend of prediction accuracy by selecting the best metabolites (metabolites with the highest effects in BayesB model), the blue line indicates the trend of prediction accuracy by selecting random metabolites.

For MP, randomly selected metabolites reduced r_ac_ but when selecting those 50% of metabolites with the highest effects in MP using the whole metabolite set, the accuracy increased to up to 0.65 for HEA (Fig 3). This trend applied to most of the traits (S9 Fig). Traits with a generally weaker r_ac_ in the MP based on all metabolites (EAR and TGW) even increased their prediction accuracy when only 10% of the most impactful metabolites were selected (S9 Fig). The model was not as robust against reduction when using metabolites instead of SNPs. This may be due to the fact that much less metabolites than SNPs are available and thus a further reduction has a stronger impact on accuracy of the model, especially with random selection. The reason for the enhancement in r_ac_ by selecting 50% of the best metabolites is probably due to the reduction of noise in the model resulting from metabolites with questionable determination quality. A study in rapeseed also showed that high prediction accuracies are possible with a reduced marker set [49]. These findings allow the consideration of using reduced and selected marker sets for GP, this way reducing computation time and costs as fewer markers have to be evaluated.

The high accuracies, especially in GP, may partly be attributed to the population design of HEB-25, which is genetically highly diverse due to the crossings with 25 different wild barley accessions. Breeding populations usually have a much smaller genetic variability [17]. Moreover, the large sample size influences the accuracies [50]. Nevertheless, the high accuracies in this study confirmed the value of applying GP in barley breeding, especially the time and cost savings are mentioned here. Results of MP indicate it as an interesting alternative to GP under certain circumstances, but according to the current status, its practical use in barley breeding is not recommendable. Metabolites as predictor variables are an attractive alternative to SNPs when no genotypic data is available, as it is the case in many orphan crop species [16]. Moreover, MP has the potential to detect metabolites involved in the expression of important agronomic traits, which might assist in unravelling the involved molecular pathways. Further research in HEB-25, like GWAS on metabolite expression, to investigate metabolite-trait associations is in progress. This promises to achieve a deeper knowledge of the complex interaction between genes, metabolites and plant physiology.

## Supporting information

S1 Table Detailed information about field trials 2011 to 2018. (XLSX)

S2 Table List of metabolites 1st sampling date. (XLSX)

S3 Table List of metabolites 2nd sampling date. (XLSX)

S4 Table Descriptive statistics of agronomic traits across the years 2011–2018. (XLSX)

S5 Table Results for genomic and metabolic prediction using the RR-BLUP model and metabolites from 1st sampling date. (XLSX)

S6 Table Descriptive statistics for the metabolites from 1st sampling date. (XLSX)

S7 Table Descriptive statistics for the metabolites from 2nd sampling date. (XLSX)

S8 Table Results for genomic and metabolic prediction using the BayesB model, including results from 2nd sampling date. (XLSX)

S9 Table Pearson's correlation coefficients between traits and metabolites and estimated metabolite effects in BayesB model, 1st sampling date. (XLSX)

S10 Table Pearson's correlation coefficients between traits and metabolites and estimated metabolite effects in BayesB model, 2nd sampling date. (XLSX)

S11 Table Results for metabolic prediction using BayesB model, phenotypic data 2017 and metabolites of 1st sampling date. (XLSX)

S12 Table Pearson's correlation coefficients between traits (phenotypic data only from 2017) and metabolites of 1st sampling date. (XLSX)

S1 Fig Distribution of markers on chromosomes. (PDF)

S2 Fig Boxplots of all traits over years and across treatments. (PDF)

S3 Fig Cross-validated prediction accuracies of traits with metabolite data using RR-BLUP and BayesB, respectively. (PDF)

S4 Fig Genomic heritability of single metabolites. (PDF)

S5 Fig Estimated effects of metabolites in BayesB model plotted against Pearson’s correlation coefficients of metabolite measurements with the agronomic trait, exemplified for HEA. (PDF)

S6 Fig Pearson’s correlations of SNP effects (a) or metabolite effects (b) estimated for respective traits in BayesB model, in comparison to correlations of trait BLUEs (c).
(PDF)

S7 Fig Scatter plot of Euclidean distances estimated with SNPs and metabolites, respectively. (PDF)

S8 Fig Variation of prediction accuracy for selected traits in BayesB through reduction of used SNP markers. (PDF)

S9 Fig Variation of prediction accuracy for selected traits in BayesB through reduction of used metabolites. (PDF)

S1 File Detailed description of field trials. (PDF)

S2 File Raw phenotype data. (XLSX)

S3 File Raw metabolite data. (XLSX)

## Abbreviations

BLUEs: *Best linear unbiased estimates*
CV: *Coefficient of variation*
EAR: *Ears per m^2^*
GC-MS: *Gas chromatography-mass spectrometry*
GEBV: *Genomic estimated breeding value*
GNE: *Grain number per ear*
GP: *Genomic prediction*
GS: *Genomic selection*
GWAS: *Genome-wide association study*
HEA: *Heading*, *flowering time*
HEI: *Plant height*
LC-MS: *Liquid gas chromatography-mass spectrometry*
MAS: *Marker-assisted selection*
MAT: *Maturity*
MNI: *Mean imputation*
MP: *Metabolic prediction*
NAM: *Nested association mapping*
NMR: *Nuclear magnetic resonance-mass spectrometry*
QTL: *Quantitative trait locus/loci*
r_ab_: *Prediction ability*
r_ac_: *Prediction accuracy*
RR-BLUP: *Ridge regression best linear unbiased prediction*
SD: *Standard deviation*
SHO: *Shooting*
SNP: *Single nucleotide polymorphism*
TGW: *Thousand grain weight*
YLD: *Grain yield*
