# Supplementary material for: Can metabolic prediction be an alternative to genomic prediction in barley?
Source: PLoS One. 2020 Jun 5;15(6):e0234052. doi: 10.1371/journal.pone.0234052 (PMC7274421; doi:10.1371/journal.pone.0234052)
Supplement: S1 Fig — (PDF) [file pone.0234052.s013.pdf]

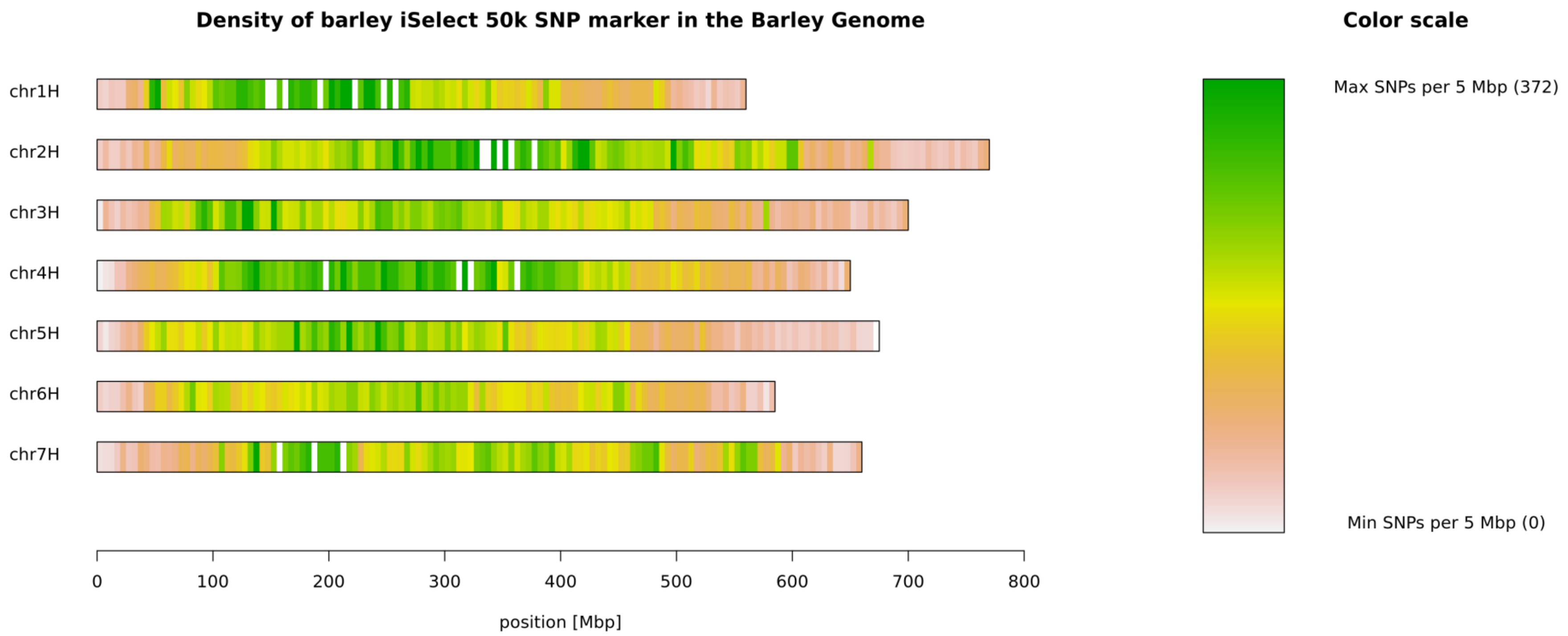

Figure S1. Distribution of markers on chromosomes. Colour indicates the density of markers per 5 Mbp. Decreasing marker density in the direction of telomeres can be observed.
