# Supplementary material for: Can metabolic prediction be an alternative to genomic prediction in barley?
Source: PLoS One. 2020 Jun 5;15(6):e0234052. doi: 10.1371/journal.pone.0234052 (PMC7274421; doi:10.1371/journal.pone.0234052)
Supplement: S2 Fig — (PDF) [file pone.0234052.s014.pdf]

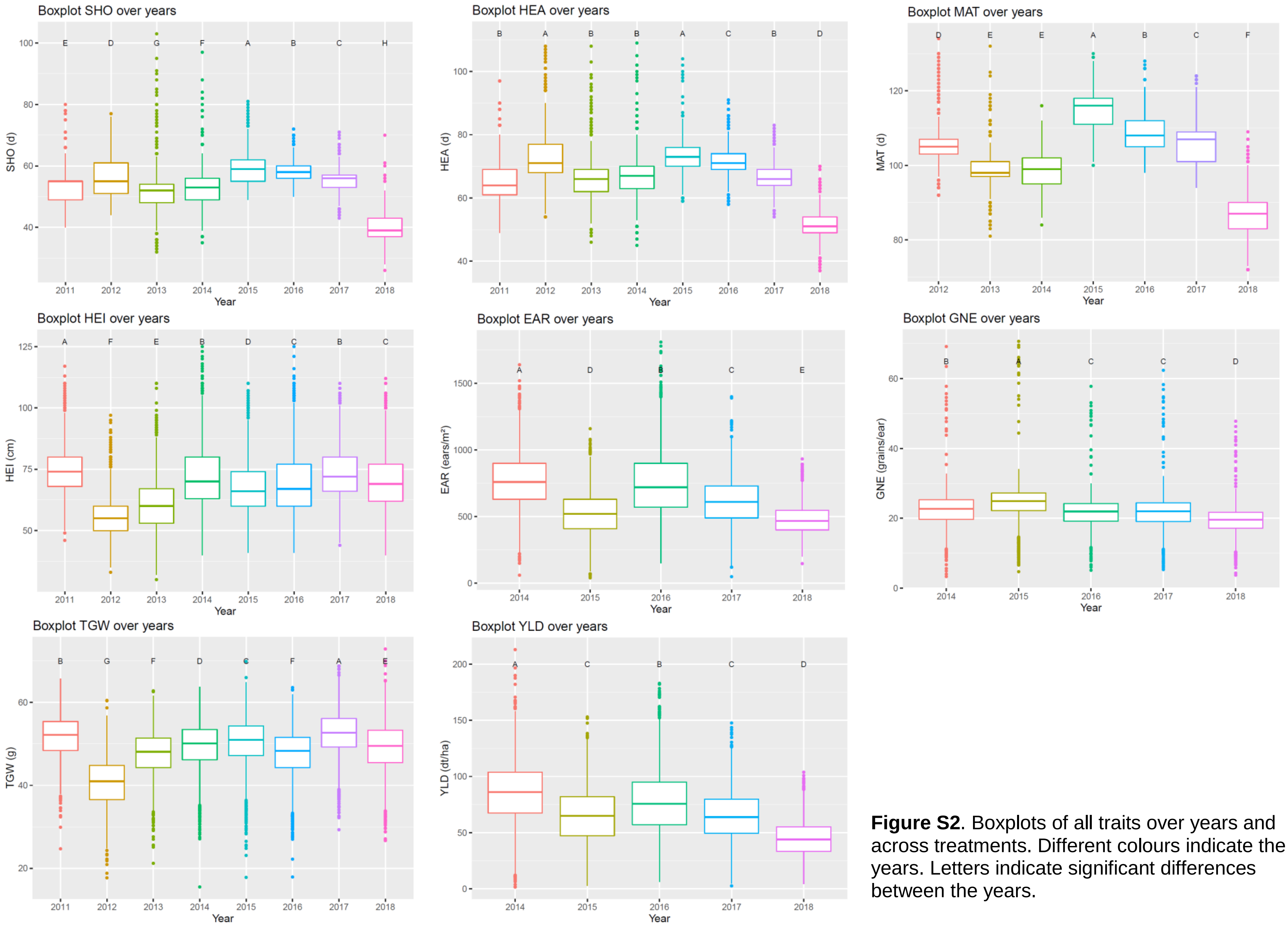


**Figure S2**. Boxplots of all traits over years and across treatments. Different colours indicate the years. Letters indicate significant differences between the years.
