# Supplementary material for: Can metabolic prediction be an alternative to genomic prediction in barley?
Source: PLoS One. 2020 Jun 5;15(6):e0234052. doi: 10.1371/journal.pone.0234052 (PMC7274421; doi:10.1371/journal.pone.0234052)
Supplement: S3 Fig — (PDF) [file pone.0234052.s015.pdf]

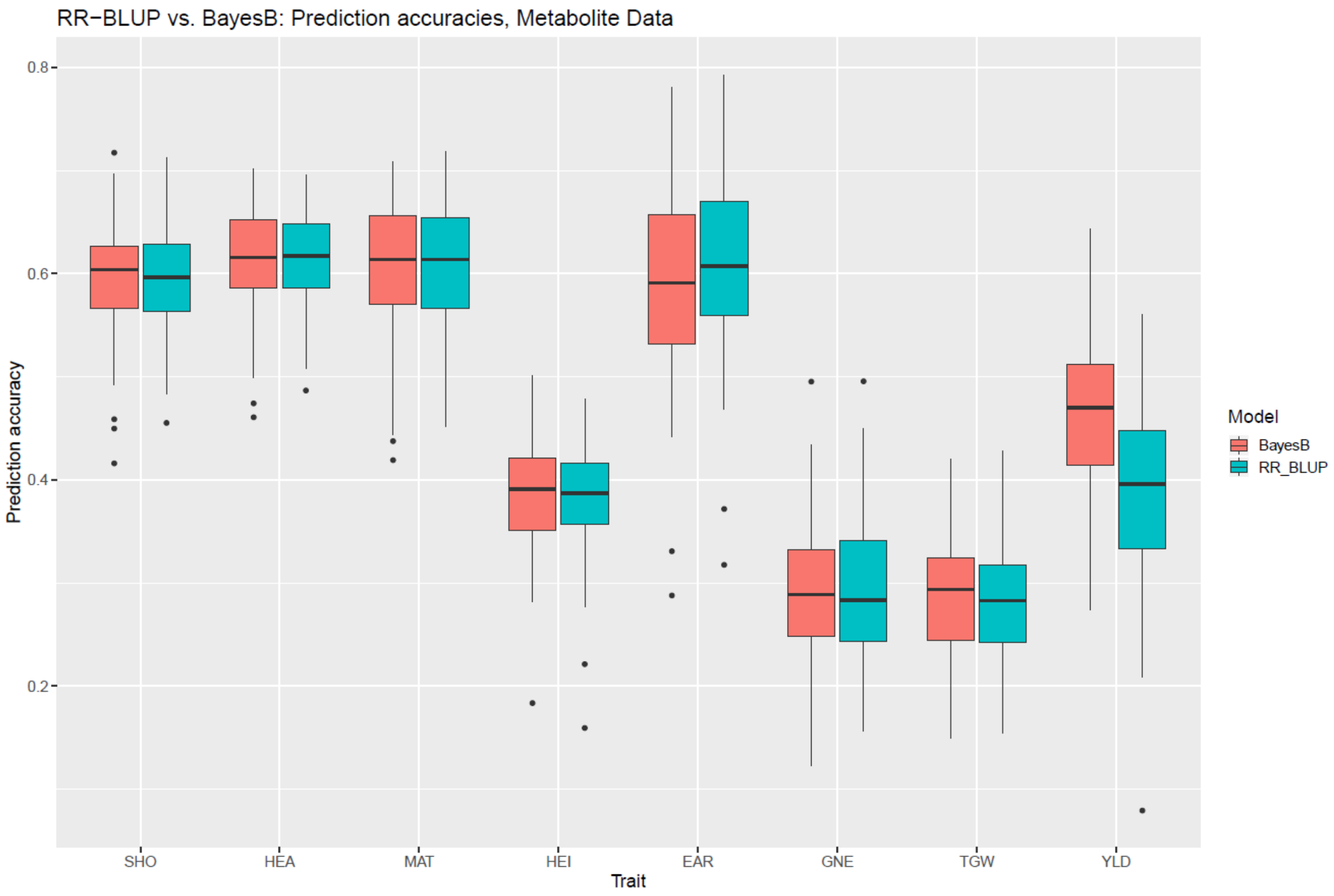

Figure S3. Cross-validated prediction accuracies of traits with metabolite data using RR-BLUP and BayesB, respectively. Boxplots contain all 100 values of the cross-validation runs. Red boxplots show results of BayesB, blue boxplots show results of RR-BLUP.
