# Supplementary material for: Can metabolic prediction be an alternative to genomic prediction in barley?
Source: PLoS One. 2020 Jun 5;15(6):e0234052. doi: 10.1371/journal.pone.0234052 (PMC7274421; doi:10.1371/journal.pone.0234052)
Supplement: S4 Fig — (PDF) [file pone.0234052.s016.pdf]

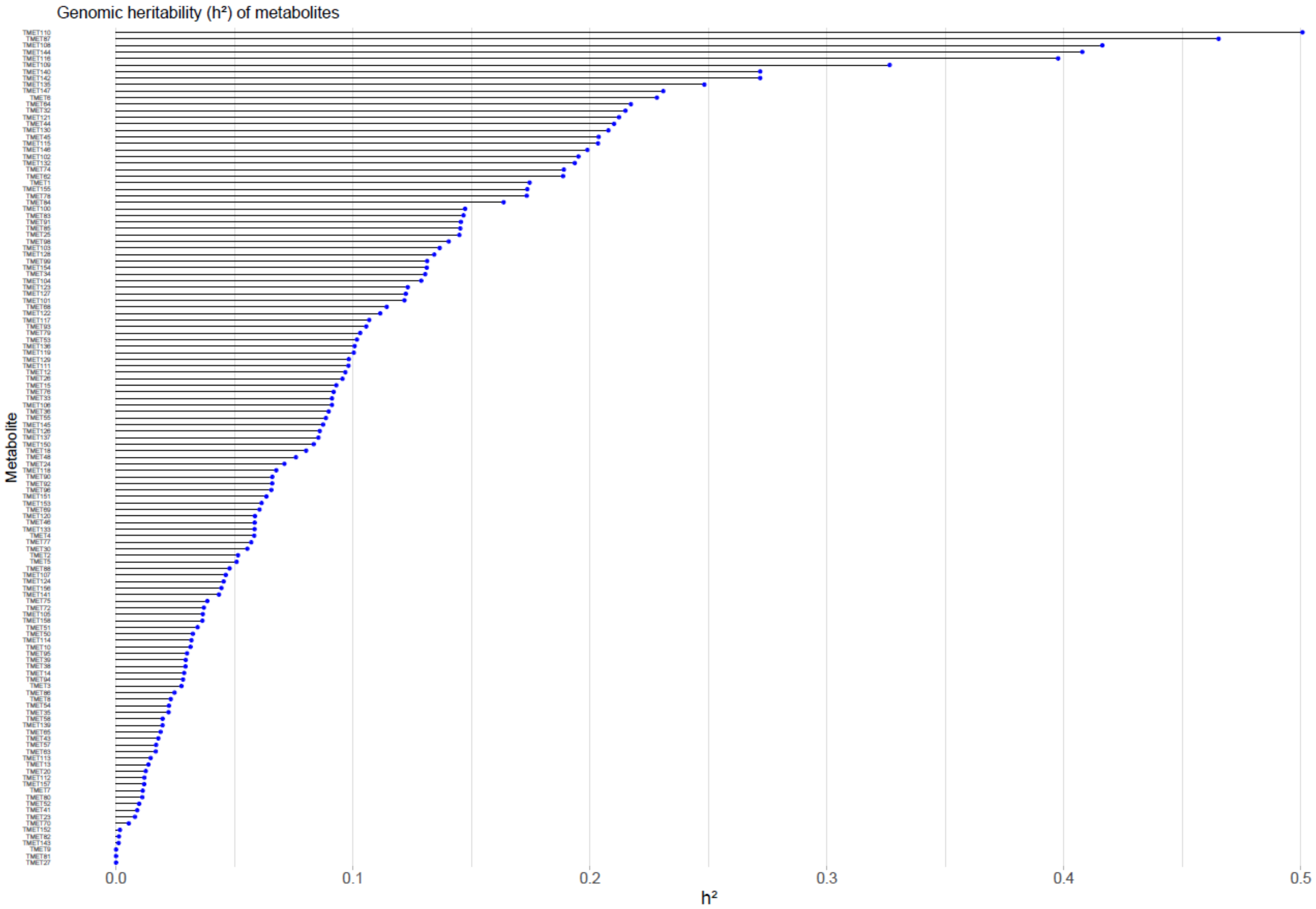


**Figure S4**. Genomic heritabilities of all metabolites. Blue dots indicate the value of $h^2$ for the respective metabolites on the y-axis.
