# Supplementary material for: Can metabolic prediction be an alternative to genomic prediction in barley?
Source: PLoS One. 2020 Jun 5;15(6):e0234052. doi: 10.1371/journal.pone.0234052 (PMC7274421; doi:10.1371/journal.pone.0234052)
Supplement: S5 Fig — (PDF) [file pone.0234052.s017.pdf]

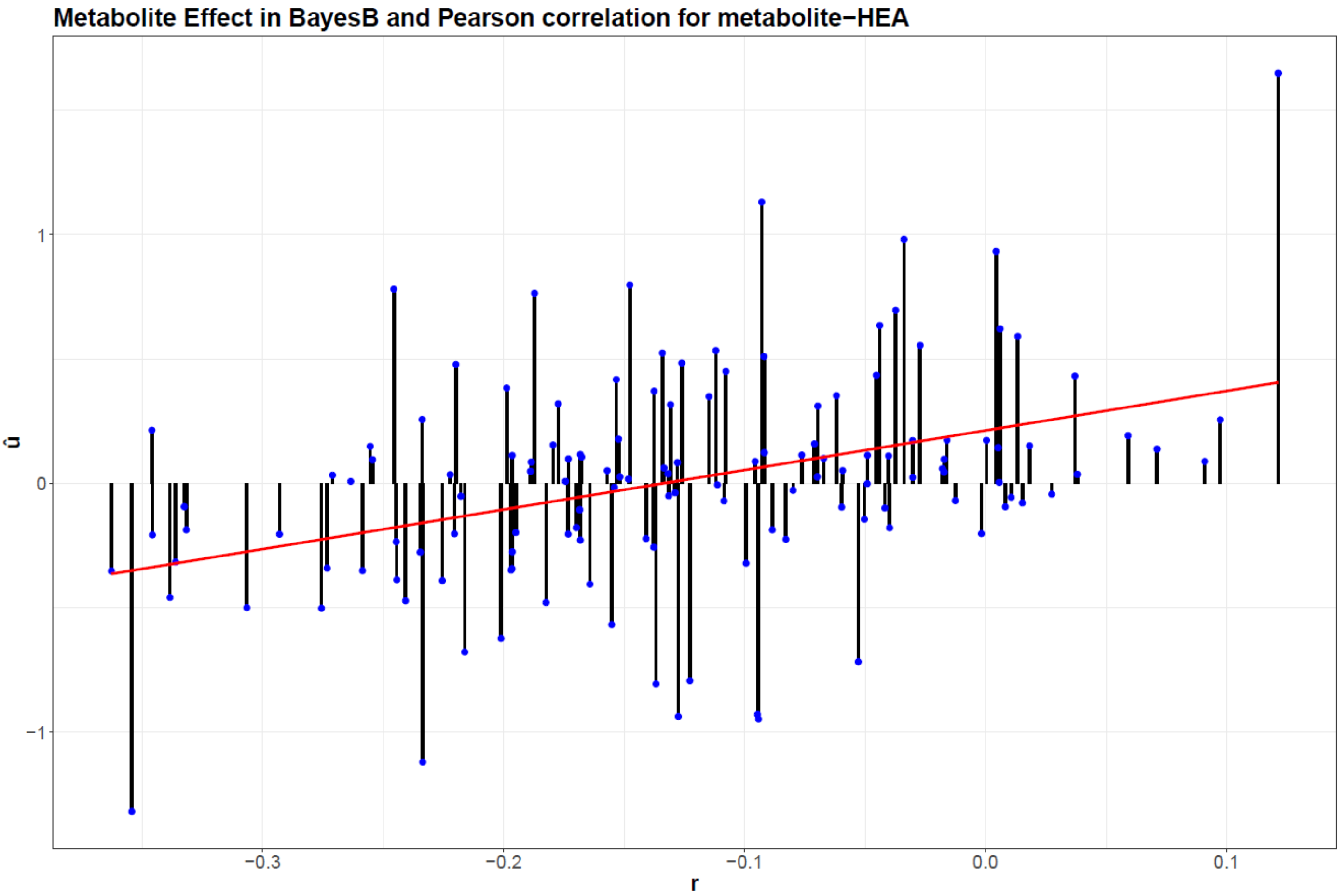

Figure S5. Estimated effects of metabolites in BayesB model (û) plotted against Pearson's correlation coefficients of metabolite measurements with the agronomic trait (r), exemplified for HEA. Regression line (red line) r = 0.39. Blue dots indicate each metabolite's û on the y-axis and r on the x axis.
