# Supplementary material for: Can metabolic prediction be an alternative to genomic prediction in barley?
Source: PLoS One. 2020 Jun 5;15(6):e0234052. doi: 10.1371/journal.pone.0234052 (PMC7274421; doi:10.1371/journal.pone.0234052)
Supplement: S6 Fig — Pearson’s correlations of SNP effects (a) or metabolite effects (b) estimated for respective traits in BayesB model, in comparison to correlations of trait BLUEs (c). (PDF) [file pone.0234052.s018.pdf]

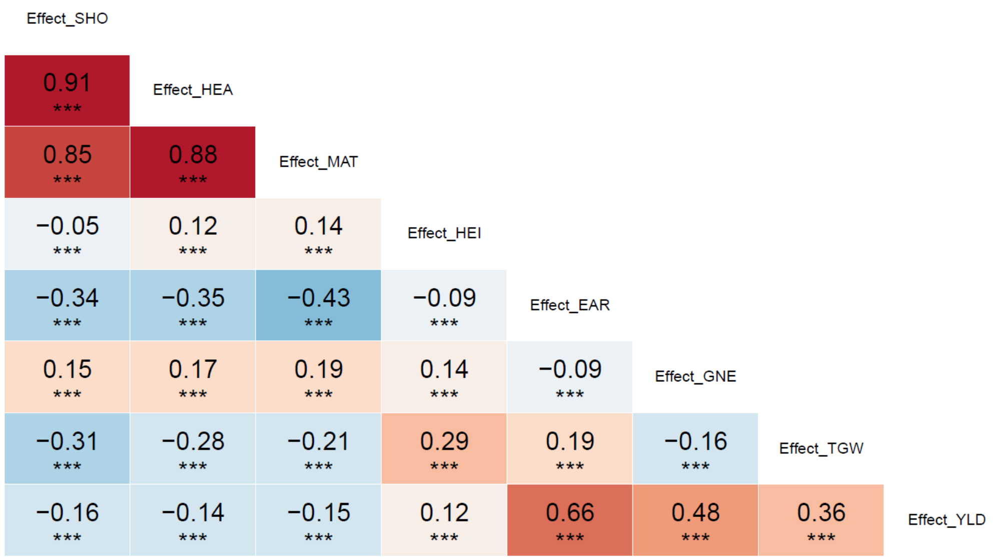

Figure S6a. Pearson's correlation of <u>SNP effects</u> estimated for respective traits in BayesB model. Significant correlation coefficients are indicated with * p < 0.05, ** p < 0.01 and *** p < 0.0001.

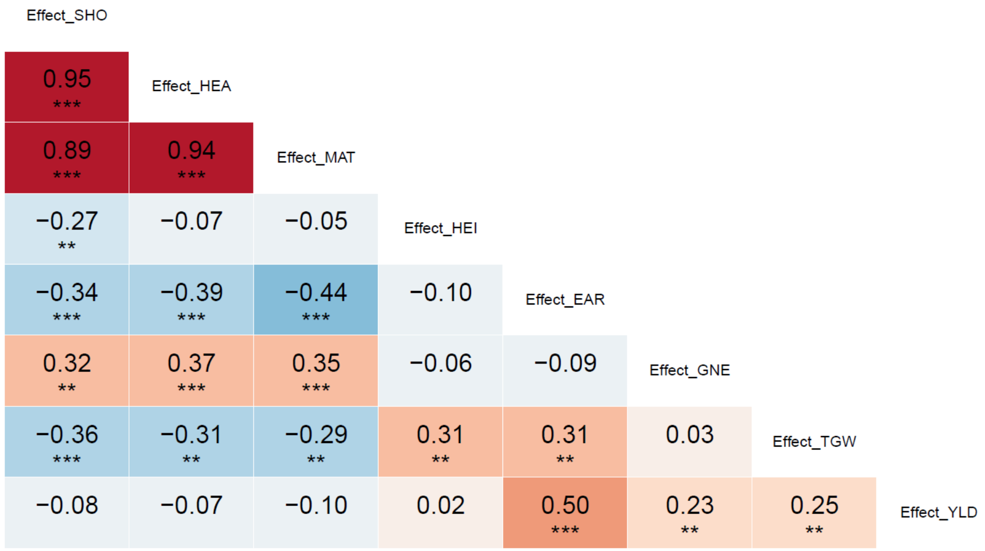

Figure S6b. Pearson's correlation of <u>metabolite effects</u> estimated for respective traits in BayesB model. Significant correlation coefficients are indicated with * p < 0.05, ** p < 0.01 and *** p < 0.0001.

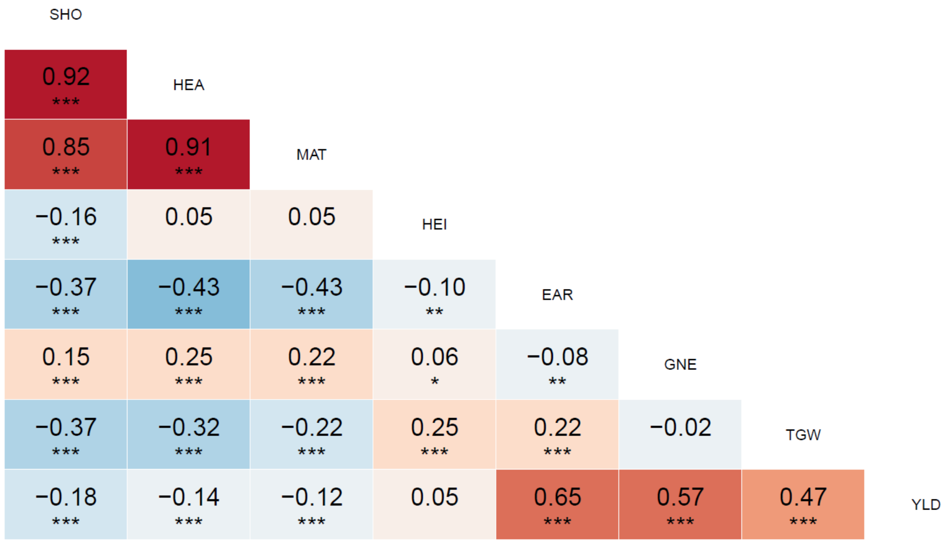

Figure S6c. Pearson's correlation of all traits based on <u>trait BLUEs</u> across years. Significant correlation coefficients are indicated with * p < 0.05, ** p < 0.01 and *** p < 0.0001.
