# Supplementary material for: Can metabolic prediction be an alternative to genomic prediction in barley?
Source: PLoS One. 2020 Jun 5;15(6):e0234052. doi: 10.1371/journal.pone.0234052 (PMC7274421; doi:10.1371/journal.pone.0234052)
Supplement: S7 Fig — (PDF) [file pone.0234052.s019.pdf]

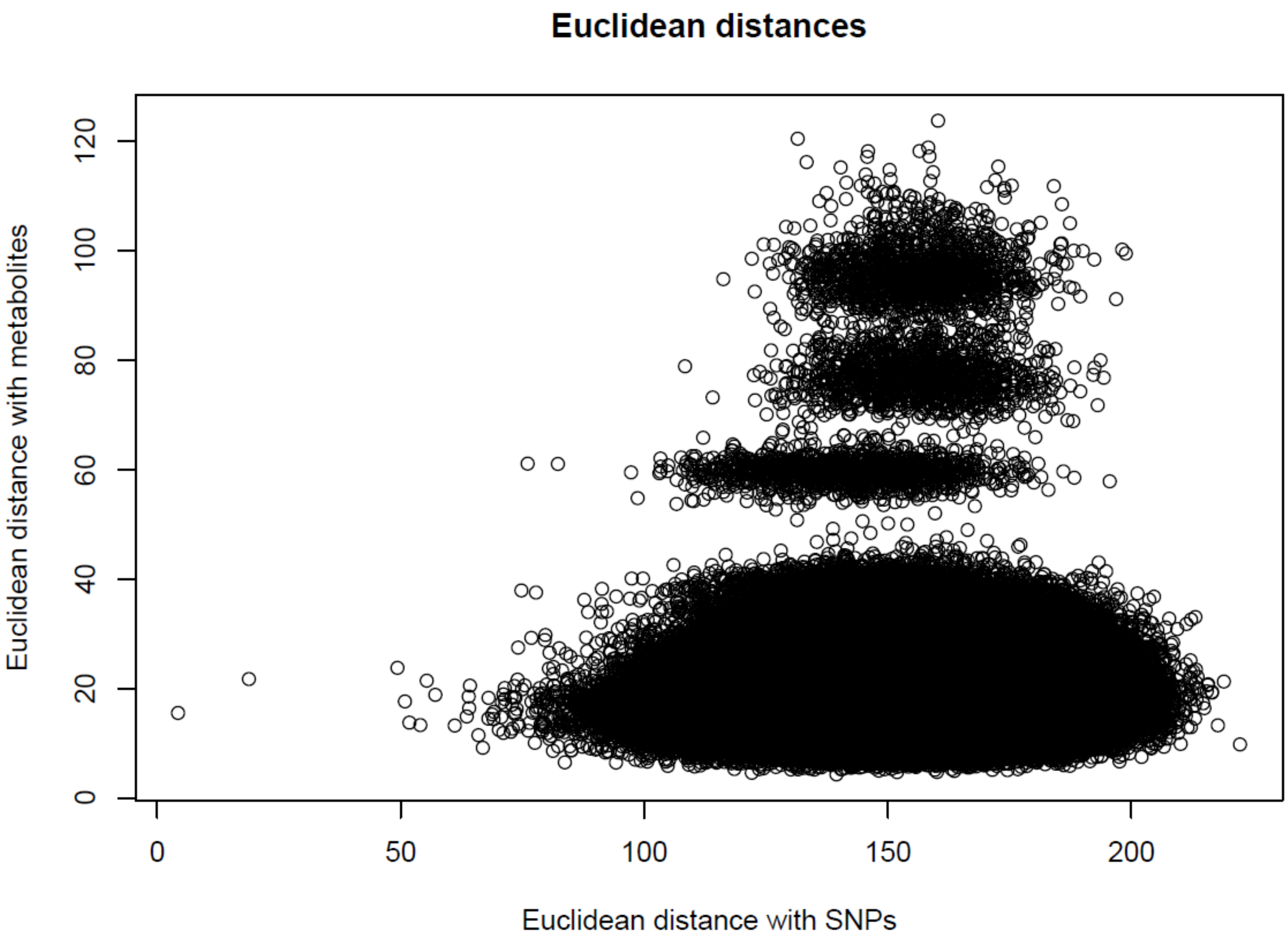

Figure S7. Scatter plot of Euclidean distances estimated with SNPs and metabolites, respectively (r = 0.04, p-value < 0.0001).
