# Supplementary material for: Can metabolic prediction be an alternative to genomic prediction in barley?
Source: PLoS One. 2020 Jun 5;15(6):e0234052. doi: 10.1371/journal.pone.0234052 (PMC7274421; doi:10.1371/journal.pone.0234052)
Supplement: S8 Fig — (PDF) [file pone.0234052.s020.pdf]

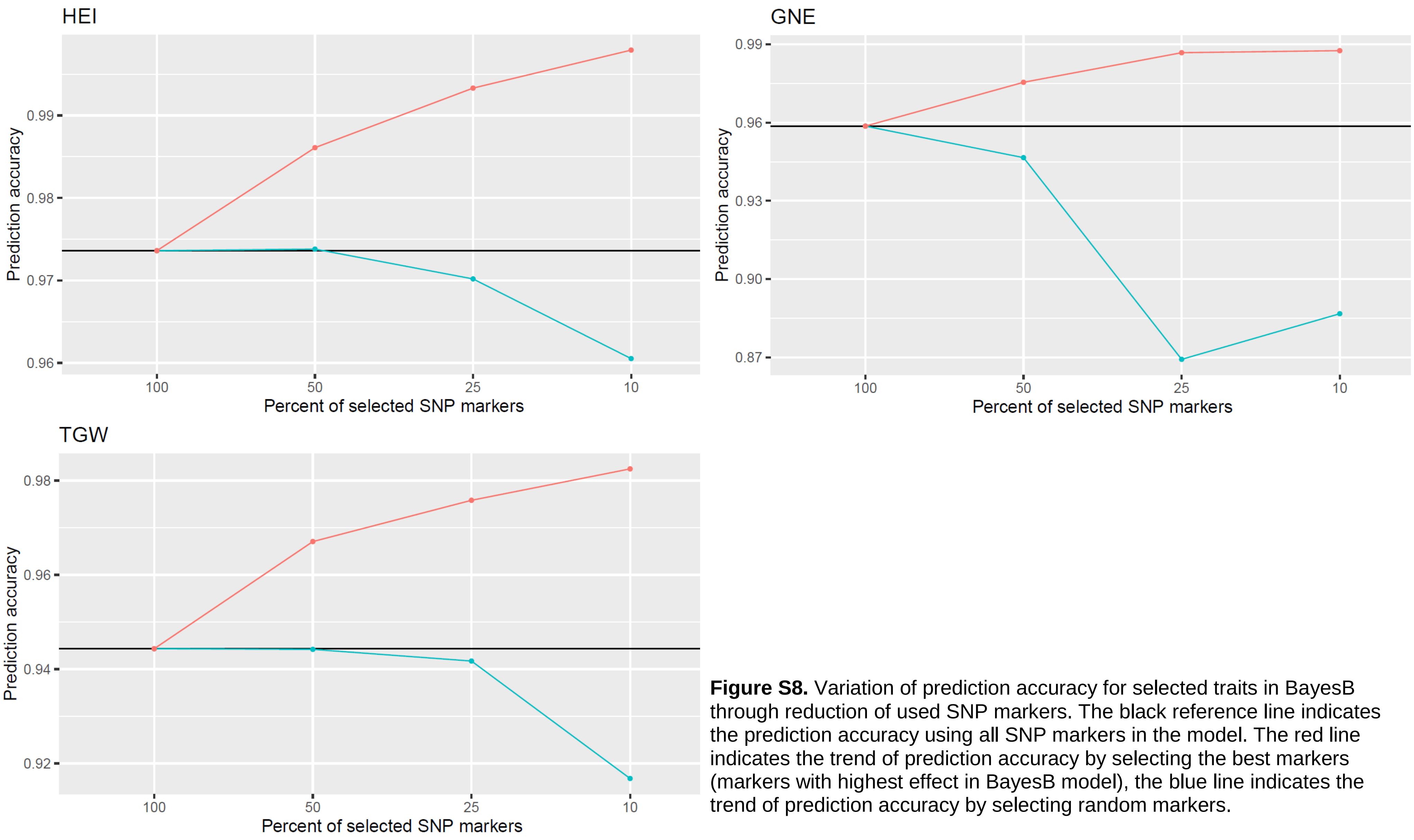

Figure S8. Variation of prediction accuracy for selected traits in BayesB through reduction of used SNP markers. The black reference line indicates the prediction accuracy using all SNP markers in the model. The red line indicates the trend of prediction accuracy by selecting the best markers (markers with highest effect in BayesB model), the blue line indicates the trend of prediction accuracy by selecting random markers.
