# Supplementary material for: Can metabolic prediction be an alternative to genomic prediction in barley?
Source: PLoS One. 2020 Jun 5;15(6):e0234052. doi: 10.1371/journal.pone.0234052 (PMC7274421; doi:10.1371/journal.pone.0234052)
Supplement: S1 File — (PDF) [file pone.0234052.s022.pdf]

Supplementary Note

Detailed description of field trials

In 2011, the field trial was conducted with selfed progenies of BC1S3 lines (so-called BC1S3:4). Sowing occurred in single to five row plots with a length of 1.50 m and a distance of 0.20 m between rows. The number of rows per HEB line and the position inside the field trial depended on the number of available BC1S3:4 seeds. Lines with seed numbers lower than ten were planted in plots with a length of 0.50 m. In 2012 and 2013, the field trials were conducted with the selfed progenies in BC1S3:5 and BC1S3:6, respectively. Two replications per HEB line, arranged in two randomized complete blocks, were cultivated in 2012 and 2013. The plots consisted of two rows (30 seeds each) with a length of 1.50 m and a distance of 0.20 m between rows. From 2014 to 2016 (generations BC1S3:7-9), the field trials were conducted in two randomized complete blocks with different nitrogen (N) treatments (N0 without fertiliser and N1 with fertiliser). Plots consisted of two rows of 50 seeds with a row length of 1.40 m and a spacing of 0.20 m between rows and 0.50 m between plots. The targeted available nitrogen in N1 was set to 100 kg N/ha in 2014 and 2015. In 2016, the fertilising target was 90 kg N/ha. The amount of added fertiliser was calculated after measuring plant available nitrogen in soil (N_{min}) as bulk sample before sowing. This resulted in fertilising the N1 block with 60 kg N/ha in 2014, 70 kg N/ha in 2015 and 60 kg N/ha in 2016. For the years 2017 and 2018 (generations BC1S3:10 and BC1S3:11) the N treatment was cancelled because of high N_{min} values in 2017 (70 kg N/ha) which made it impossible to generate distinctions in N supply with realistic fertiliser quantities in N1. Therefore, the trial was changed into two different fungicide treatments (Fun0 without and Fun1 with fungicide). In 2018, the plants were sown in three row plots with a length of 1 m and a distance of 15 cm between rows.
